# First report on human T-lymphotropic virus 1 infection in a group of transgender women

**DOI:** 10.3389/fpubh.2024.1459707

**Published:** 2024-10-30

**Authors:** Megmar Aparecida dos Santos Carneiro, Mykaella Cristina Araújo Margarida, Bruno Vinícius Diniz e Silva, Ágabo Macedo da Costa e Silva, Márcia Alves Dias de Matos, Karlla Antonieta Amorim Caetano, Sheila Araújo Teles, Antonio Carlos Rosário Vallinoto, Regina Maria Bringel Martins

**Affiliations:** ^1^Institute of Tropical Pathology and Public Health, Federal University of Goiás, Goiânia, Brazil; ^2^Faculty of Nursing, Federal University of Goiás, Goiânia, Brazil; ^3^Laboratory of Virology, Institute of Biological Sciences, Federal University of Pará, Belém, Brazil

**Keywords:** HTLV-1, transgender women, prevalence, RDS, Brazil

## Abstract

**Introduction:**

There is a lack of data on human T-lymphotropic virus 1 (HTLV-1) infection among transgender women (TGW). Therefore, this study estimated the prevalence of HTLV-1 infection in a group of TGW in Brazil.

**Methods:**

A cross-sectional study was conducted with 235 TGW in Goiânia City, Central Brazil. Respondent-driven sampling was used for recruitment. All participants were interviewed, and serum samples were tested for anti-HTLV-1/2 using an enzyme-linked immunosorbent assay (ELISA; Murex HTLV-I + II, DiaSorin, Dartford, United Kingdom). Seropositive samples were submitted for confirmation by Western blot (WB; MP Diagnostics HTLV BLOT 2.4 test, MP Biomedicals, Germany).

**Results:**

The majority of participants were young (≤ 25 years old), self-declared mixed or brown ethnicity, single, had attended high school, and had a monthly income above US$ 255 (R$ 1,000,00; nearly the minimum wage in Brazil at the time). Most reported earlier age at first sex and various risk behaviors for sexually transmitted infections (STIs). Three TGW were anti-HTLV-1/2 positive by ELISA and were subsequently positive for HTLV-1 by WB, giving a crude HTLV-1 seroprevalence of 1.3%; 1.0% (95% CI: 0.0–1.9) after being weighted by RDS Analysis Tool (RDSAT). The HTLV-1 seropositive TGW reported high-risk sexual behaviors. In addition, one of them also engaged in injecting drug use.

**Conclusion:**

These data indicate the circulation of HTLV-1 in TGW in Goiânia City, Central Brazil. Given the alarming estimates of high-risk sexual behaviors, there is an urgent need to intensify health programs targeting this population to control and prevent HTLV-1 and other STIs effectively.

## Introduction

The human T-lymphotropic virus 1 (HTLV-1) causes a lifelong infection, which is endemic in diverse world regions, including South America. It is associated with several diseases, such as adult T-cell leukemia/lymphoma (ATL) and HTLV-1-associated myelopathy/tropical spastic paraparesis (HAM/TSP), besides other inflammatory diseases (1).

This virus is transmitted by unprotected sexual intercourse, injecting drugs or transfusion/transplantation of contaminated blood/tissue, and vertically from mother to child, mainly by breastfeeding. Regarding its sexual transmission, risk factors, including earlier age at first sexual intercourse, higher number of sexual partners, and unprotected sexual intercourse increase the risk of HTLV-1 infection (1–3).

Transgender people have a gender identity or gender expression that is different from their assigned birth sex. Transgender women (TGW) are disproportionately affected by human immunodeficiency virus (HIV) and other sexually transmitted infections (STIs) (4). TGW are a stigmatized population, often marginalized, with high levels of transphobia, violence, and discrimination in different spheres of socialization, such as family, school, work, and health services. This unfavorable social context experienced by TGW often relates to their involvement in sex work, use/abuse of psychoactive substances, and other risk behaviors, contributing to their vulnerability to HIV and other STIs, including HTLV-1 infection (5–7). Nonetheless, there is a lack of data on HTLV-1 infection among transgender women. Thus, studies on HTLV-1 epidemiology in this socially marginalized population are needed to understand the burden of this infection and to contribute for preventive actions. Therefore, this study aims to estimate the prevalence of HTLV-1 in a group of TGW in Brazil.

## Methods

This cross-sectional study was conducted among TGW in Central Brazil, who were recruited using the respondent-driven sampling (RDS) method in Goiânia City (the capital of the State of Goiás), Central Brazil, between April 2018 and August 2019. In this study, as previously described in a study investigating human papillomavirus (HPV) infection (8), the recruitment process started with a non-random selection of five key members of the TGW population denominated as “seeds” who received three numbered referral coupons to recruit members of their social network. Each participant also received three coupons to recruit new members from the target population. The inclusion criteria for participation were: to self-identified themselves as transgender women and to present a valid RDS coupon to the study recruiter at enrollment; there were no age limitations, and injectable drug users were not excluded from the study. TGW were recruited regardless of their health status. During the interviews, TSW who appeared to be under the influence of drugs and alcohol (drunk or incoherent), in a manner that rendered it difficult for them to understand the research questions, were excluded from the study.

Initially, all participants were informed of the objectives and methodology of the study. Eligible TGW aged 18 or older signed the Free and Informed Consent Form. Those younger than 18 years of age signed the Free and Informed Assent Form. The protocol for this study was approved by the Ethics Committee for Human Research of the Federal University of Goiás (protocol numbers CAAE 77481417.5.0000.5083 and 2.358.818). Trained personnel interviewed the participants in a private room using a structured script containing questions to obtain sociodemographic data, risk behaviors, and HIV status. Then, a blood sample (10 mL) was collected from each participant.

All serum samples were tested for anti-HTLV-1/2 antibodies using an enzyme-linked immunosorbent assay (ELISA) (Murex HTLV-I + II, DiaSorin, Dartford, United Kingdom). ELISA seropositive samples were also tested for confirming and differentiating the presence of antibodies to HTLV-1 and HTLV-2 using a Western blot (MP Diagnostics HTLV BLOT 2.4 test, MP Biomedicals, Germany). HTLV Blot 2.4 uses a combination of recombinant HTLV-1/2 proteins and HTLV-1 viral lysate to improve sensitivity. This assay also uses HTLV type-specific recombinant envelope protein (gp46-1 and gp46-2) to discriminate viral types. Results of MP HTLV Blot 2.4 were interpreted according to the stringent criteria provided by the manufacturer. Briefly, HTLV-1 seropositivity was defined as reactivity to gag (p19 with or without p24) and two env (GD21 and rgp46-I) bands. HTLV-2 seropositivity was defined as reactivity to gag (p24 with or without p19) and two env (GD21 and rgp46-II) bands. Samples that were reactive to both gag (p19 and p24) and env (GD21) were defined as HTLV seropositive but were considered untypable. Any other patterns of specific bands that did not meet the above criteria were considered as indeterminate. Absence of bands were interpreted as negative.

Positive samples were tested for HIV co-infection using a four-generation ELISA for the simultaneous detection of HIV-1 p24 antigen and anti-HIV-1/2 antibodies (BIOLISA HIV 1/2/O ANTIGEN/ANTICORPO, Bioclin, Quibasa, Belo Horizonte, Brazil). All assays were conducted according to the respective manufacturer’s instructions.

The collected data were entered into EpiData software version 3.1 and were subsequently exported for analysis. RDS Analysis Tool (RDSAT) version 7.1.76^1^ was used to generate prevalence estimates with 95% confidence interval (CI) using 15,000 bootstrap iterations. The network size of each participant was measured by the number of TGW whom they reported that they knew.

## Results

Based on the RDS sampling method, a total of 235 TGW was included in this study: five were seeds and 230 recruits. Regarding their sociodemographic characteristics, nearly 50% of the participants were young (≤ 25 years old, 49.5%). Most were mixed (or brown; 51.7%), single (87.7%), had attended high school (10–12 years of formal education, 63.1%), and had a monthly income above US$ 255 (R$ 1,000,00; nearly the minimum wage in Brazil at the time; 67.5%; Table 1).

**Table 1:** Characteristics of the studied TGW in Central Brazil.

Variable	N	Unweighted %	Weighted by RDSAT^a^	
			%	95% CI
Age (years)				
≤ 25	137	58.3	49.5	37.7–65.3
> 25	98	41.7	50.5	34.7–62.3
Skin color/race (self-reported)^b^				
White	43	18.5	20.5	11.8–30.0
Brown	129	55.4	51.7	40.8–63.7
Black	41	17.6	18.9	11.7–27.5
Asian	15	6.4	6.1	1.4–11.0
Indigenous	5	2.1	2.8	0.1–7.0
Marital status^b^				
Single	209	89.3	87.7	79.0–95.7
Married/stable relationship	22	9.4	12.0	3.9–21.0
Separated/divorced/widow	3	1.3	0.3	0.0–1.1
Schooling (years)				
≥ 13	25	10.6	10.6	3.8–17.8
10–12	147	62.6	63.1	51.0–72.6
≤ 9	63	26.8	26.3	17.2–39.2
Monthly income				
> R$ 3,000 (> U$ 767)	70	29.8	23.3	16.3–31.3
R$ 1,001–3.000 (U$ 256–767)	114	48.5	44.2	35.7–53.1
≤ R$ 1,000 (≤ U$ 255)	51	21.7	32.5	23.4–41.3
History of incarceration^b^				
No	177	75.6	81.3	74.9–87.0
Yes	57	24.4	18.7	13.0–25.1
Age of first sex (years)				
≤12	52	22.1	28.4	19.4–37.8
13–15	89	37.9	36.3	28.3–44.7
>15	94	40.0	35.3	27.3–43.9
History of forced sex				
No	121	51.5	61.0	51.1–69.3
Yes	114	48.5	39.0	30.7–48.9
Transactional sex				
No	22	9.4	20.3	10.2–30.3
Yes	213	90.6	79.7	69.8–89.8
Number of sexual partners in the last week				
≤1	53	22.6	44.8	27.5–61.4
2–10	60	25.5	22.7	15.9–31.3
11–20	41	17.4	9.8	5.8–15.3
>20	81	34.5	22.7	12.9–33.3
Condom use in the last intercourse^b^				
No	155	67.7	65.1	56.2–73.6
Yes	74	32.3	34.9	26.4–43.8
Receptive anal intercourse^b^				
No	8	3.4	3.1	0.6–6.6
Yes	226	96.6	96.9	93.4–99.4
Insertive anal intercourse^b^				
No	47	20.1	36.6	25.1–48.2
Yes	187	79.9	63.4	51.8–74.9
Group sex^b^				
No	37	15.8	27.0	17.2–36.6
Yes	197	84.2	73.0	63.4–82.8
Chemsex^b^				
No	58	24.8	35.2	24.0–46.1
Yes	176	75.2	64.8	53.9–76.0
Sex with drug user partner^b^				
No	25	10.7	27.9	14.4–43.0
Yes	209	89.3	72.1	57.4–85.6
History of STI				
No	116	49.4	59.8	50.7–68.5
Yes	119	50.6	40.2	31.5–49.3
TWH^b^				
No	172	83.5	88.8	82.1–93.3
Yes	34	16.5	11.2	6.7–17.9

RDSAT, RDS Analysis Tool; CI, confidence interval; STI, sexually transmitted infections; TWH, transgender women living with HIV (self-reported).

a Population-based estimates are based on analysis in RDSAT, which is adjusted for personal network sizes and recruitment patterns.

b Missing data are not shown.

Few TGW reported receiving a blood transfusion (*n* = 20) and injection drug use (IDU, *n* = 9). Regarding sexual behaviors (Table 1), the majority of participants reported their first sexual intercourse before 16 years old (64.7%). A history of forced sex was noted in 39% of TGW. Approximately one-third had more than 10 sexual partners during the week preceding the interview (32.5%). Almost 70% affirmed no use of condoms in their last sexual intercourse. Most TGW reported lifetime transactional sex (79.7%) and practiced receptive (96.9%) and insertive (63.4%) anal sex. Also, they practiced group sex (73.0%), chemsex or sexualized drug use (64.8%), and sex with drug users (72.1%). Of the total, 40.2% had a history of STIs, and 11.2% were transgender women living with HIV (TWH). A history of incarceration was observed in nearly 20% of the TGW studied.

Among the 235 TGW who were tested for anti-HTLV-1/2, three were anti-HTLV-1/2 positive by ELISA and were subsequently positive for HTLV-1 by WB, giving a crude HTLV-1 seroprevalence of 1.3%; 1.0% (95% CI: 0.0–1.9) after being weighted by RDSAT.

Characteristics of the HTLV-1 seropositive TGW are shown in Table 2. Their ages ranged from 24 to 37 years; two self-declared white, and one brown. They had 10 to 12 of formal education, a monthly income between US$ 562 and 767, and also reported in-country migration. The two younger (MT-174 and MT-213) reported receiving industrial liquid silicone application, previous incarceration, and a history of STIs. One of these (MT-213) also engaged in injecting drug use. All seropositive TGW denied having received a blood transfusion or gender-affirming surgery. However, they reported high-risk sexual behaviors, such as earlier age at first sexual intercourse (7 to 11 years old), history of forced sex, transactional sex, receptive and insertive anal intercourse, group sex, chemsex and sex with drug user partner, as well as unprotected sexual intercourse with multiple partners (>10) in the last 12 months. There were no HTLV-1 and HIV co-infected participants.

**Table 2:** Characteristics of HTLV-1-positive transgender women in Central Brazil.

Characteristics	MT-121	MT-174	MT-213
Age (years)	37	25	24
Ethnicity (self-reported)	White	Brown	White
Naturality	GO	CE	PA
In-country migration	Yes	Yes	Yes
Education (years)	12	12	10
Monthly income (U$)	562	767	767
Gender-affirming surgery	No	No	No
Industrial liquid silicone application	No	Yes	Yes
History of blood transfusion	No	No	No
Previous incarceration	No	Yes	Yes
Ever used illicit injecting drug	No	No	Yes
Age of first sex	7	11	10
History of forced sex	Yes	Yes	Yes
Transactional sex (lifetime)	Yes	Yes	Yes
Receptive anal intercourse (lifetime)	Yes	Yes	Yes
Insertive anal intercourse (lifetime)	Yes	Yes	Yes
Use of condoms (last 12 months)	Never	Sometimes	Sometimes
Number of sexual partners (last 12 months)	10	1,000	3,000
Group sex (lifetime)	Yes	Yes	Yes
Chemsex (lifetime)	Yes	Yes	Yes
Ever had sex with drug user partner	Yes	Yes	Yes
History of STIs	No	Yes	Yes
HIV co-infection	No	No	No

GO, Goiás; CE, Ceará; PA, Pará; STIs, sexually transmitted infections; HIV, human immunodeficiency virus.

## Discussion

This is the first study to investigate the prevalence of HTLV-1 infection among TGW. The sociodemographic characteristics of the studied population were similar to those reported in other Brazilian TGW populations (7, 9), such as mostly young, self-reported as brown, single, with 10–12 years of formal education or less, and a monthly income above US$ 255 (R$ 1,000,00; nearly the minimum wage).

Nearly half of the enrolled TGW reported a history of forced sex. Similarly, other Brazilian TGW experienced sexual violence (10, 11), and this was associated with depression (12). As observed in other studies (9, 10, 13), risk behaviors such as transactional sex, engaging in unprotected sexual intercourse, and having multiple sexual partners were frequent among the TGW investigated, corroborating with data shown in a systematic review and meta-analysis estimating the prevalence of sexual behaviors among the US transgender population (14). In this study, chemsex, as well as a history of STIs, group sex, and sex with drug users were highly frequent among participants. It is noteworthy that a multicentric study of Brazilian TGW revealed an association of chemsex with self-reported STI, more than five sexual partners, and condomless anal sex (6). Moreover, according to Luz et al. (15), presenting at least one active STI was associated with having had group sex in the last year and with chemsex. Taken together, these findings reinforce the alarming vulnerability of TGW to STIs and the importance of urgent need prevention strategies targeting this specific population.

The present study provides the first set of data describing the seroprevalence of HTLV-1 in a group of TGW in Central Brazil. Although no similar reports are available for direct comparison, the adjusted estimative of 1.0% for anti-HTLV-1 found in these TGW was similar to that reported in patients with pulmonary tuberculosis in Central Brazil (0.99%) (16) but higher than that observed in local blood donors (0.09%) (17). Nevertheless, this seroprevalence falls within the confidence interval reported for Brazilian pregnant women (0.32%; CI 95%: 0.19–1.54) (18), as well as for those reported in populations at risk for STIs, such as female sex workers in Pará, Northern region (1.8%; CI 95%: 0.0–5.8) (19), and men who have sex with men (MSM) in Campinas, Southeast region (1.5%; 95% CI: 0.5–3.0) (20); it was, however, slightly higher than that found among MSM in Central Brazil (0.7%; 95% CI: 0.4–0.9) (21).

Despite most TGW studied engaged in high-risk sexual behaviors, no HTLV-1 and HIV co-infected participants were observed. Previously, in another study in Central Brazil, a low prevalence of coinfected patients was found (0.79%) (22). Regarding studies conducted in other regions of Brazil, rates of HTLV-1/HIV-1 co-infection varying from 0.8% (Londrina and surrounding region, Paraná, Southern region) to 17.1% (Salvador, Bahia, Northeast region) (23, 24) were reported. In a later large-scale study also conducted in the state of Bahia, HTLV was detected in 2.4% of the HIV-positive samples (*n* = 42/1,733); most of them were HTLV-1 reactive (88.1%) (25). In this continental country, differences in its regional endemicity, ethnic origin of the population, risk behaviors, and study designs are probably the reasons for these differences in co-infection rates.

The three anti-HTLV-1 positive TGW reported in-country migration, a detail that aligns with findings from another Brazilian study, in which TGW also experienced family rejection, discrimination, and violence (10). These authors observed an association between in-country migration and HIV infection among TGW in Northeastern Brazil. Additionally, they reported that the main reason that led them to migrate was the need to look for a job opportunity and to improve their quality of life. Conversely, in-country migration may increase their existing vulnerability. Therefore, given the increased mobility and vulnerability of TGW, they have a higher risk of HIV infection and probably of other STIs, including HTLV-1 infection. As reported elsewhere (26), HTLV-1 infection is a public health problem, especially in vulnerable populations, such as migrants, and understanding health inequities is essential for implementing effective measures to reduce the burden of its associated diseases.

HTLV-1 is transmitted primarily through direct contact via cell-containing bodily fluids, including blood, and thus, injecting drug use is a risk factor for HTLV-1 infection (1). It can be considered one of the modes of transmission among this TGW group since one HTLV-1 seropositive (MT-213) engaged in injecting drug use. Notably, all seropositive TGW reported sex with drug users, as well as chemsex, which may impact HIV and other STIs vulnerability (6). Of note, all but one had a previous history of STIs. In line with other studies (1–3), a history of unprotected sex, an earlier age at first sexual intercourse, and a higher number of partners increase the risk of HTLV-1 transmission. In fact, these behaviors were reported by the three anti-HTLV-1 positive TGW in addition to other high-risk sexual behaviors (transactional sex, receptive and insertive anal intercourse, and group sex).

Considering the alarming vulnerability of TGW to STIs and the importance of effective prevention strategies targeting this specific population, studies have highlighted the challenges TGW faces in accessing health services, education, and formal jobs (7, 27, 28). Addressing these challenges and overcoming stigma and discrimination, health strategies for preventing STIs are urgently required to achieve the World Health Organization’s goal of global elimination of HIV, viral hepatitis, and STIs, including HTLV-1, as a public health threat by 2030 (29).

This study features some limitations. First, the data were collected in Goiânia City, Central Brazil, and they may not represent the general Brazilian TGW population. However, their sociodemographic characteristics are similar to those reported in other Brazilian TGW studies (7, 9). Second, some results are subject to response biases as a limitation of face-to-face interviews. Data collection based on self-report may produce recall bias. However, some strategies were implemented to minimize potential biases, including previously trained interviewers and private interview room. Third, vertical transmission of HTLV-1 by breastfeeding among TGW studied could not be analyzed for lack of this information. Finally, whole blood samples from the anti-HTLV-1 positive TGW were unavailable to detect HTLV proviral DNA. Therefore, all ELISA reactive samples were confirmed by WB and typed as HTLV-1. Furthermore, the small sample size investigated may have limited the number of HTLV-1 seropositive TGW in this study, and thus, this limited our statistical power to identify the demographic and behavioral characteristics associated with HTLV-1. Despite these limitations, this study provided the first data on HTLV-1 among TGW. Nevertheless, studies with a large sample of TGW are needed to estimate a more accurate prevalence of HTLV-1 and to identify factors associated with this infection among TGW to formulate public health policies addressing its prevention.

## Conclusion

These results reveal for the first time not only the circulation of HTLV-1 among TGW in Goiânia, Central Brazil, but also alarming estimates of high-risk behaviors, particularly sexual behaviors. Therefore, specific health programs that focus on diagnosing, controlling, and preventing HTLV-1 and other STIs should be implemented for TGW, mitigating social barriers and promoting the implementation of appropriate interventions for adequate access to health services.

## Data Availability

The raw data supporting the conclusions of this article will be made available by the authors, without undue reservation.

## Footnotes

^1^http://rds-analysis-tool.software.informer.com/versions/
